# In Commemoration of Dr. Mahdokht Pourmansour, an Outstanding Specialist in the Production of the B.C.G. Vaccine in Iran

**DOI:** 10.52547/ibj.3903

**Published:** 2023

**Authors:** Fatemeh Bardestani, Ehsan Mostafavi

**Affiliations:** Department of Epidemiology and Biostatistics, Research Centre for Emerging and Reemerging Infectious Diseases, Pasteur Institute of Iran, Tehran, Iran

## INTRODUCTION

With the aim of providing community health, the Pasteur Institute of Iran was established in 1920 as the oldest medical and health research institution in Iran. In addition to conducting research relevant to infectious diseases and manufacturing biological products, this institute has significantly contributed to the prevention and control of communicable diseases for more than a century. 

As an elder in the institute, Dr. Mahdokht Pourmansour, a physician and specialist in microbiology and laboratory science, was a prominent researcher at the Pasteur Institute of Iran. During four decades of her service to the institute, she has performed remarkable activities, particularly in the development of the Bacille Calmette-Guérin vaccine to prevent tuberculosis. In this biography paper, her scientific life and services are reviewed.

**Early** **life**

Mahdokht Pourmansour was born on January 7^th^, 1935 in Tehran, the capital city of Iran. She attended Mehregan and Asadi elementary schools in Tehran from 1940 to 1946. During the time periods 1946-1952, she continued her high school education at Nizam Vafa in Ahvaz and obtained her high school diploma at Azarm high school in Tehran. From 1952 to 1958, she received her doctorate in medicine from the University of Tehran^[^^1^^]^. During the years 1967-1968, she attended the Pasteur Institute of Paris on a scholarship from the Pasteur Institute of Iran. After returning to the country in 1974, she presented the courses she took in France and obtained Ph.D. degree in clinical laboratory science at the University of Tehran. Her Ph.D. dissertation was written on the development of a cholera vaccine in laboratory animals^[^^2^^]^.

**What** **makes her famous** **?**

**Fig. 1:**
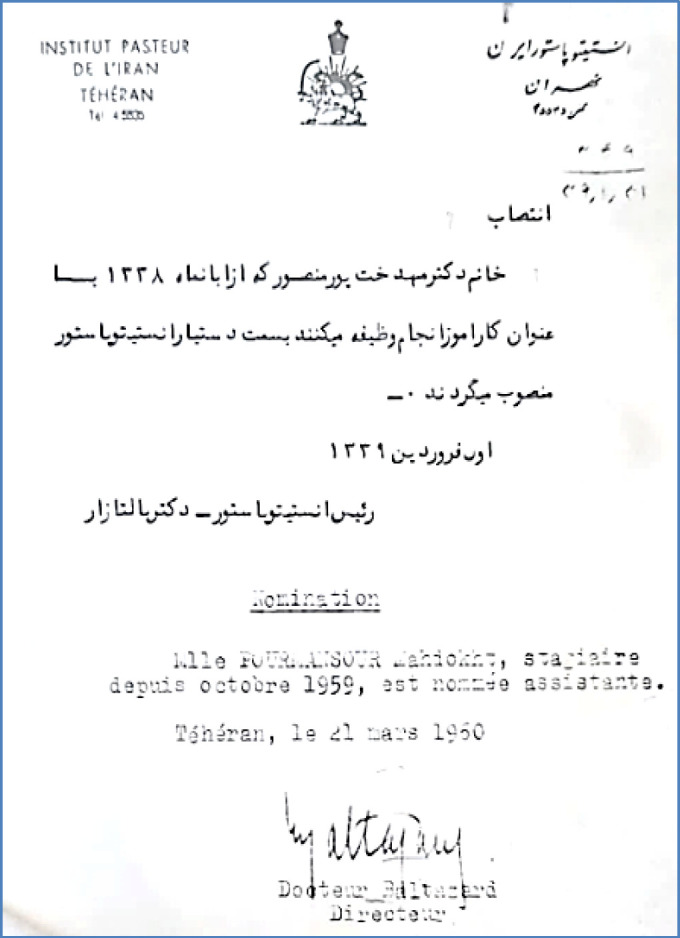
Appointment of Dr. Mahdokht Pourmansour as an assistant with Dr. Marcel Baltazard, the former director of the Pasteur Institute of Iran, 1960

Mahdokht Pourmansour worked at Shirin High School in Tehran from 1954 to 1959 following her diploma graduation. After obtaining the medical degree, she worked as an assistant in the Obstetrics and Gynecology Department at maternity hospital for mothers and infants for two years (1959-1960). Simultaneously, in 1960, she started working as an assistant at the Pasteur Institute of Iran (Fig. 1). 

**Fig. 2:**
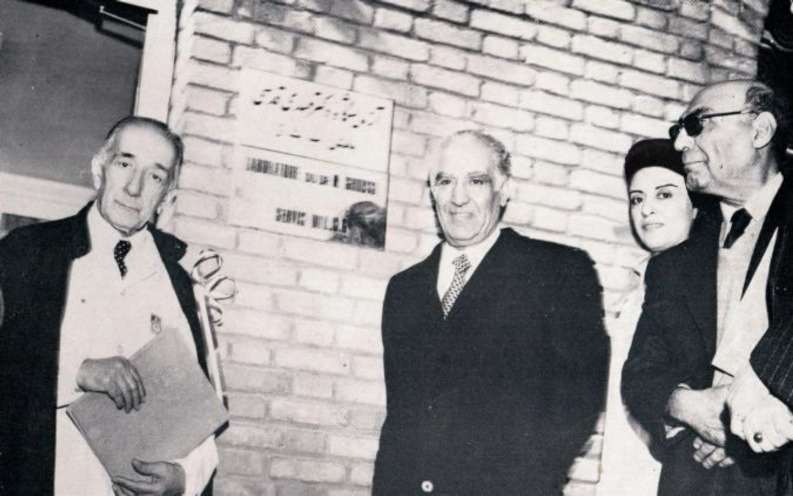
From right: Dr. Sabbar Mirza Farman Farmaian, the former director of the Pasteur Institute of Iran, Dr. Mahdokht Pourmansour, the head of the Department of B.C.G., Dr. Manouchehr Iqbal, Professor of the Infectious Disease at the University of Tehran, and Dr. Mehdi Ghodsi, the senior consultant and former head of the Department of B.C.G., the Pasteur Institute of Iran, 1973

Dr. Pourmansour became the director of the Department of Microbiology at the same Institute from 1964 to 1971, and then started working at the Department of B.C.G. under the supervision of Dr. Mehdi Ghodsi (Fig. 2). Over these years, she also managed the Department of tuberculosis for two years. 

**Fig. 3:**
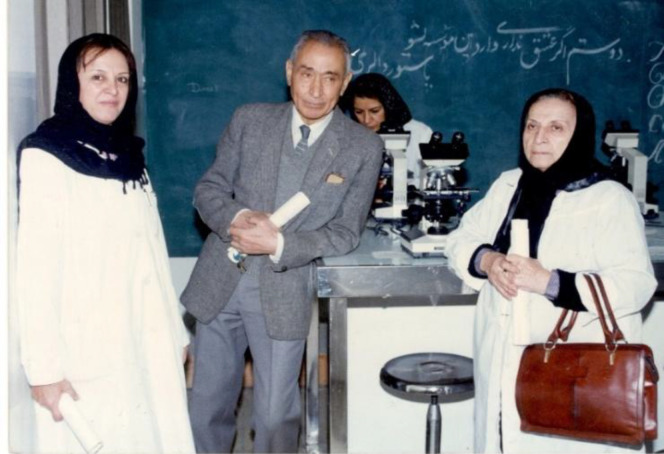
From left to right: Dr. Mahdokht Pourmansour, Dr. Rasul Pournaki, the former director of the Virology Department, and Dr. Dorreh Al-Sadat Tabatabaei, the former director of the Tuberculosis Department, the Pasteur Institute of Iran,1981^[^^3^^]^.

**Fig. 4:**
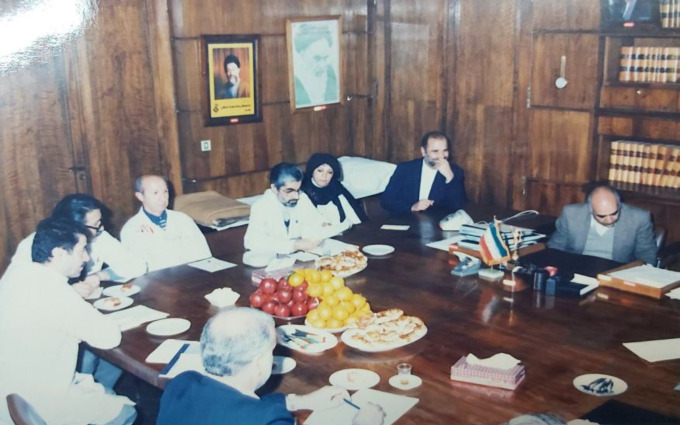
Meeting of the managers of the Pasteur Institute of Iran. From the right: Dr. Hassan Hakimi (Deputy Director of Production), Dr. Ahad Milaninia (the former director of the Pasteur Institute of Iran), Dr. Mahdokht Pourmansour, Dr. Ahmad Fayaz, Dr. Manouchehr Mohammadi, Mohammad Ali Bahavar, Dr. Mehdi Asmar (Deputy Director of Research), anonymous, 1988

In addition to serving as the head of the Microbiology Department at the Pasteur Institute of Iran from 1991 to 1994, Dr. Pourmansour also managed the Microbiology Research Group. Her responsibility as the director of this department was supervising five departments of this research group, including Bacteriology, Parasitology, Medical Entomology, Mycology, Virology, and Rabies (Figs. 3 and 4).

As a senior research scientist and expert, she established the Department of Biological Product Control at the Pasteur Institute of Iran in 1986. She was a member of the Scientific Publications (1988) and Expert Councils (1987-1992) at the same Institute during her service. Dr. Pourmansour was a senior advisor and the first chair of the Scientific Council of the Pasteur Institute of Iran. She has cooperated with the World Health Organization (WHO) as an expert since 1970. She was also responsible for the implementation and policy-making of research in the fight against infectious diseases at the Pasteur Institute of Iran in addition to other universities and the Ministry of Health (1992). In the same year, she also was in charge of setting up the Center for Communicable Disease Control (CDC) at the Pasteur Institute of Iran. 

After 38 years of service, Dr. Mahdokht Pourmansour retired on March 26^th^, 1994. During the years 1994-2004, she continued to serve in the research planning and contribution to the development of the Pasteur Institute of Iran as a research consultant and member of the scientific council. 

**Career** **highlights**

**About the production of the B.C.G. vaccine in the Pasteur Institute of Iran**

"B.C.G." (Bacillus Calmette-Guérin) is the initials of two French scientists’ names (Albert Calmette and Camille Guérin), the researchers of the Pasteur Institute of Paris who produced this vaccine for the first time. The content of the vaccine is a live attenuated bacillus of bovine tuberculosis. In this symmetrical work and during World War I, the Pasteur Institute of Paris kept the original and reliable strain of B.C.G. in the Tuberculosis Department and donated it to all vaccination institutes, especially the Pasteur Institute members around the world. After World War II, in 1946, in the presence of the director of the Pasteur Institute of Iran, Dr. Marcel Baltazard, and after traveling several scientists of the Pasteur Institute of Paris to Iran to cooperate with technical and scientific fields, it was decided to begin the production of B.C.G. vaccine in Iran. The first B.C.G. strain was transferred to Iran by plane in the spring of 1947 by Dr. Georges Blanc, the director and founder of the Pasteur Institute of Casablanca (Morocco) who discovered the anti-typhoid vaccine. The Pasteur Institute of Iran succeeded to produce this vaccine in a small laboratory with the minimum facilities. The first B.C.G. vaccine was prepared in October 1947 and then successfully used by the experts of the Pasteur Institute of Iran to vaccinate many children in the Tehran Municipal Nursery. Gradually, by increasing the production capacity at the Pasteur Institute of Iran, it was injected into health centers in Tehran and then other health centers in the country. This vaccine was prepared by Dr. Mehdi Ghodsi and Dr. Mahdokht Pourmansour with care and compliance with all scientific and technical principles. The first batch of B.C.G. vaccine was produced in the form of 2 ml lyophilized vials and placed at the disposal of health and medical centers under the cold - controlled cold temperature, and after being dissolved in the solution made by the Pasteur Institute of Iran, the vaccine was inoculated into the arms of infants. Children were vaccinated twice with this vaccine. The production project of B.C.G. was carried out with the support of the WHO. In 1950, to revise the production of this vaccine, a contract was made with the Pasteur Institute of Paris, and until 1952, vaccination was performed by intracutaneous injection, and since 1953, the vaccine was injected by the intradermal route. The average annual production of B.C.G. vaccine was three million units, and the liquid vaccine was utilized until 1976. Then, with the provision of suitable equipment and place, the lyophilized vaccine was prepared. The stability of a lyophilized vaccine in adverse thermal and environmental conditions is much more than a liquid vaccine^[^^4^^]^.

**Production of cholera vaccine in the Pasteur Institute of Iran**

In the first fifty years of the establishment of the Pasteur Institute of Iran, at least five cholera epidemics occurred in Iran. Hence, this institute prepared a large number of cholera vaccines. In studies conducted by the teams of the Pasteur Institute of Iran, it was found that the water of some Tehran's aqueducts was contaminated with cholera-causing bacteria. To prevent cholera epidemics in Tehran, the Pasteur Institute of Iran distributed the mineral water from springs around Tehran in closed containers to the public and also disinfected the drinking water of Tehran^[^^4^^]^.

During the cholera outbreaks in Iran and eastern neighboring countries, about 400,000 doses of cholera vaccine were daily prepared at the Pasteur Institute of Iran, and about 24 million doses of cholera vaccine were produced in a limited time. During the cholera outbreak of 1927, due to the high demand of the country for cholera vaccine and insufficient production of the vaccine by the Pasteur Institute of Iran, the part of cholera vaccine required for the country was purchased from Germany. The cholera outbreak occurred again in 1959-60. At this time, The Ministry of Health provided some of the required equipments from Germany to develop vaccine production at the Pasteur Institute of Iran. The Pasteur Institute of Iran managed to prepare about 7-10 thousand doses of the cholera vaccine and then delivered it to the Ministry of Health of Iran. In 1960, during the cholera outbreak in Afghanistan and Pakistan, the Pasteur Institute of Iran produced 5.9 million doses of cholera vaccine for these two countries. In the same year, countries like India, Iraq, Georgia, and Azerbaijan requested to receive the cholera vaccine from Iran. In 1955, when another cholera outbreak swept the country, the Pasteur Institute of Iran and the Razi Institute prepared millions of cholera vaccines. The amount of cholera vaccine production at the Pasteur Institute of Iran in 1970 reached 23 million doses, of which 15 million doses were provided for the Ministry of Health, and 28 million doses were sent to East Pakistan, Turkey, Saudi Arabia, and Ethiopia. Thus, Pasteur Institute of Iran played a central role in controlling cholera epidemics in this region^[^^4^^]^.

**Scientific** **activities**

Dr. Pourmansour's specialized fields of activity involved the search of TB and the production of the B.C.G. vaccine. Researchers of the Pasteur Institute of Iran have been preparing B.C.G. liquids according to scientific and technical principles since 1946. Dr. Pourmansour had the responsibility to keep producing this vaccine since 1971, under the supervision of Dr. Mehdi Ghodsi, the former head of the B.C.G. Department who produced the first dry B.C.G. vaccine sample in 1976^[^^5^^]^. This vaccine produced by the support of WHO, was injected intradermally into all babies in the country. Also, by performing the tuberculin skin test, they studied the emergence of resistance in the vaccinated individuals^[^^6^^]^. Several experts from the Centre for Communicable Diseases at the Ministry of Health and health experts throughout the country, have benefited from her knowledge in the field of TB and the B.C.G. vaccine over the years.

One of the duties of the Pasteur Institute of Iran since its first years of establishment in 1920, was the production of the cholera vaccine^[^^5^^,^^6^^]^. The cholera vaccines produced by the Pasteur Institute of Iran were exported to the countries such as Afghanistan, Pakistan, India, Iraq, Turkey, Saudi Arabia, Ethiopia, Georgia, and Azerbaijan, and the institute has played a dominant role in controlling cholera epidemics in these regions. Another activity of Dr. Pourmansour during the managing the Microbiology Department was to supervise the mass production of high-quality cholera vaccines^[^^5^^]^. 

Dr. Pourmansour also investigated different aspects of salmonellosis. She actively contributed to a comprehensive study attributing to the prevalent salmonellae in Iran over the years 1970 to 1974^[^^7^^]^. In 1982, she participated in an international project to determine the geographical distribution of Salmonella serotypes, worldwide^[^^8^^]^. At the same time, she also carried out a project to evaluate the relationship between listeriosis and fetal deaths in Iran. One of her latest research was the study of *Legionella pneumophila* infection of Tehran hospital water samples in 1997^[^^9^^]^. She also performed several researches on mycoplasma and antiserum production. In 2012, Dr. Mahdokht Pourmansour was honored as one of the top two microbiologists in the country by the Iranian Microbiology Association and received an award from the National Congress of Microbiology held at Tehran.

**Scientific** **trips**

In 1957, Dr. Pourmansour spent times at the Statens Serum Institute in Copenhagen, Denmark and the Pasteur Institute of Paris to prepare several samples of the lyophilized B.C.G. vaccine to compare the characteristics of the lyophilized B.C.G. vaccine produced at the Pasteur Institute of Iran with the international standards. She travelled to many countries such as Hungary (Tuberculosis Congress, 1983), the USA (Immunology and Allergy Congress, Chicago, 1992) and France (Listeriosis Congress, 1983, 1985 and 1991) to participate in scientific meetings. Dr. Pourmansour had a journey to Alexandria, Egypt and Ethiopia in 1970 to teach the vaccine lyophilization techniques. She went to Syria in 1972 at the invitation of the WHO to set up a laboratory for endemic diseases in this country.

**Books and** **papers**

**Fig. 5:**
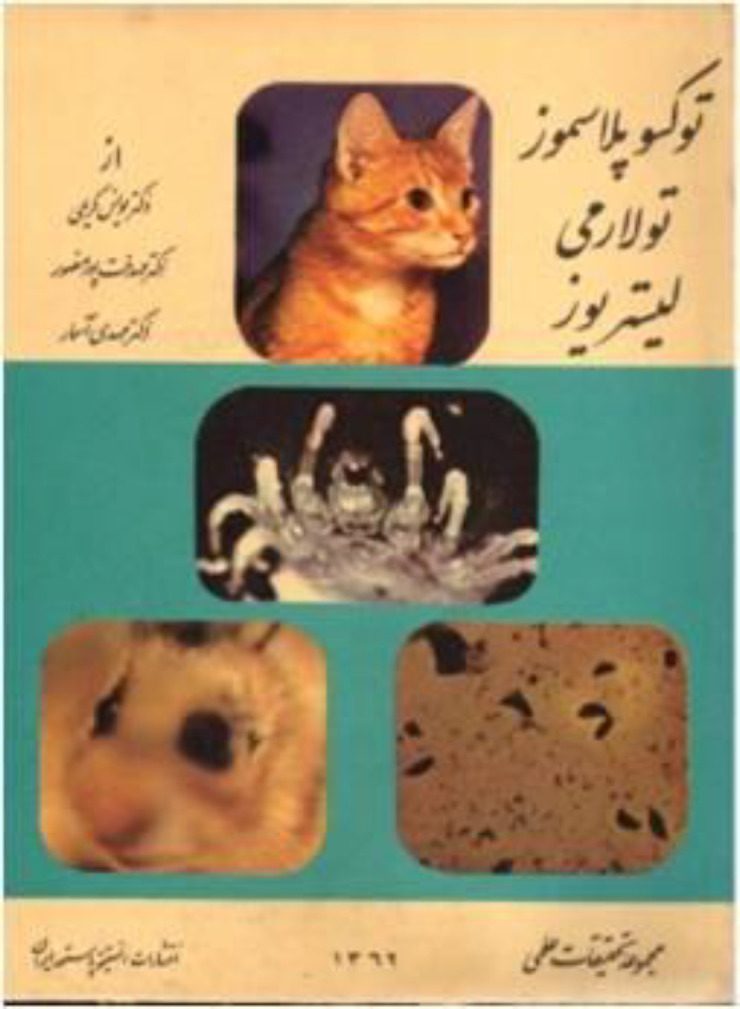
Toxoplasmosis, Tularemia, and Listeriosis, a publication of the Pasteur Institute of Iran, 1983, Written by Dr. Younes Karimi, Dr. Mahdokht Pourmansour, and Dr. Mehdi Asmar

The book "Thirty Years of Continuous Care of the B.C.G. Strain in Iran", published in Persian and French in 1978, is one of Dr. Pourmansour's works^[^^10^^]^. She wrote a chapter in the book "Toxoplasmosis, Tularemia and Listeriosis" in 1983^[^^11^^]^ (Fig. 5) and a chapter "The Application of Tuberculin and B.C.G." from the book "Tuberculosis" in 1987^[^^12^^]^.

## Conclusion

Dr. Mahdokht Pourmansour can be a successful role model for young researchers in Iran. Despite having a medical degree and the chance to continue her clinical work, she chose the field of public health and worked at the Pasteur Institute of Iran to play a significant role in the production of B.C.G. and cholera vaccines to control these diseases in the country. She was strict in overseeing the correct execution of the affairs and narrates that "I tried to send all the employees of the Department of B.C.G. to attend the specialized courses at the Pasteur Institute of Paris or the 

Copenhagen Institute in Denmark to maintain discipline in working in a standard international production unit”. She advises the future-making youth in the country by recommending effort and hope: "We worked hard to build a better future for you; you should do this for posterity. You should be careful not to waste your life's opportunity as far as you can, and you should act in such a way that at the end of your service, you will be satisfied with your performance".
